# Evaluating pediatric and adult simulated fluids solubility: Abraham solvation parameters and multivariate analysis

**DOI:** 10.1007/s11095-021-03123-8

**Published:** 2021-10-25

**Authors:** Mariana Guimarães, Martin Kuentz, Maria Vertzoni, Nikoletta Fotaki

**Affiliations:** 1 Department of Pharmacy and Pharmacology, University of Bath, Bath, UK; 2 Institute of Pharma Technology, University of Applied Sciences and Arts Northwestern Switzerland, 4132 Muttenz, Switzerland; 3 Department of Pharmacy, National and Kapodistrian University of Athens, Athens, Greece; 4 Centre for Therapeutic Innovation and Department of Pharmacy and Pharmacology, University of Bath, Claverton Down Bath, Bath, BA2 7AY UK

**Keywords:** Abraham solvation parameters, Biorelevant, Multivariate analysis, Pediatric, Solubility

## Abstract

**Purpose:**

To understand drug solubilization as a function of age and identify drugs at risk of altered drug solubility in pediatric patients. To assess the discrimination ability of the Abraham solvation parameters and age-related changes in simulated media composition to predict *in vitro* drug solubility differences between pediatric and adult gastrointestinal conditions by multivariate data analysis.

**Methods:**

Differences between drug solubility in pediatric and adult biorelevant media were expressed as a % pediatric-to-adult ratio [Sp/Sa (%)]. Solubility ratios of fourteen poorly water-soluble drugs (2 amphoteric; 4 weak acids; 4 weak bases; 4 neutral compounds) were used in the analysis. Partial Least Squares Regression was based on Abraham solvation parameters and age-related changes in simulated gastrointestinal fluids, as well as their interactions, to predict the pediatric-to-adult solubility ratio.

**Results:**

The use of Abraham solvation parameters was useful as a theory-informed set of molecular predictors of drug solubility changes between pediatric and adult simulated gastrointestinal fluids. Our findings suggest that the molecular solvation environment in the fasted gastric state was similar in the pediatric age-groups studied, which led to fewer differences in the pediatric-to-adult solubility ratio. In the intestinal fasted and fed state, there was a high relative contribution of the physiologically relevant surfactants to the alteration of drug solubility in the pediatric simulated conditions compared to the adult ones, which confirms the importance of an age-appropriate composition in biorelevant media.

**Conclusion:**

Statistical models based on Abraham solvation parameters were applied mostly to better understand drug solubility differences in adult and pediatric biorelevant media.

## Introduction

Age-related developmental changes have the potential to affect the composition of gastrointestinal (GI) fluids, which can ultimately impact on the biorelevant solubility of drugs (1). Compound solubility and dissolution are important variables regarding oral absorption, particularly for poorly water-soluble drugs. Therefore, the development of biorelevant media to study drug solubility and dissolution has been a landmark in biopharmaceutical research (2). More recently, age-appropriate biorelevant media have been developed (1). Their use is fundamental for the *in vitro* assessment of oral drug performance of poorly-water soluble compounds, especially in younger population cohorts in comparison to adults. Therefore, biopharmaceutical research could make use of these tools to evaluate “what if” scenarios regarding drug solubilization and to establish risk assessment of efficacy and safety of oral pediatric drugs.

In a recent study, the Abraham solvation parameters were calculated from the chemical structure of diverse compounds to predict the ratio of solubility enhancement (log (SE)) in Fasted state simulated intestinal fluid (FaSSIF) compared to aqueous buffer solubility at pH 6.5 (3). Solubility enhancement by a colloidal phase is essentially a solvation process for which the critical molecular characteristics are captured by the Abraham solvation parameters; these molecular predictors have been described in the literature and may characterize the transfer of a solute from one phase to another (4–6). Accordingly, selection of such predictors for any solvation-related application has the advantage of being theory-informed as opposed to a purely empirical selection of molecular descriptors in a quantitative model (4–6). Such theory-informed regression models are of interest to gain a better molecular understanding of drug solubilization as well as to enable *in silico* predictions with comparatively lower overfitting risk of a given dataset.

The aim of this work was to use Abraham solvation parameters to interpret differences between drug solubility in pediatric and adult biorelevant media of poorly water-soluble drugs that have various physicochemical properties, in terms of their lipophilicity (logP) and ionization profiles (*i.e.* weak bases, weak acids, ampholytes and neutral compounds).

## Materials and Methods

The Partial Least Squares Regression (PLS-R) analysis was conducted for 14 poorly soluble compounds (biopharmaceutics classification system (BCS) class II and IV compounds) for which solubility was measured in adult and pediatric biorelevant media representative of gastric and intestinal GI fluids in the fasted and fed state (1, 7)*.* Molecular modeling was conducted using the Absolv prediction module (2016.1 release), ACD software Percepta (Advanced Chemistry Development Inc., Canada). The molecular structures were entered as simplified molecular input line entry specification (SMILES) and the software makes use of predictive algorithms for every parameter that were developed from a dataset of 5′700 compounds with experimental values using a set of molecular fragmental descriptors. The calculated Abraham solvation parameters include *A* and *B* that hold for hydrogen-bonding acidity, and basicity, respectively. The parameter *S* represents dipolarity/polarizability, which is the ability of a solute to stabilize a neighboring dipole by its capacity of orientation and induction interactions. Moreover, *E* is the excess molar refraction descriptor, and *V* holds for the McGowan characteristic volume of a solute. More details on these molecular parameters can be inferred from a review by Poole *et al*. (2009)(4). The Abraham molecular solvation parameters are commonly used to describe the logarithm of any solvation-related property (SP) that is expressed as a linear free energy relationship of the solute properties, as presented in Eq. 1 (3).

1 $${\log}\;\text{SP}=\text{c}+\text{eE}+\text{sS}+\text{aA}+\text{bB}+\text{vV}$$

where the capital letters in Eq. 1 stand for the Abraham solvation parameters (4) and *c*, *e*, *s*, *a*, *b* and *v* are adjustable coefficients.

**Fig. 1:**
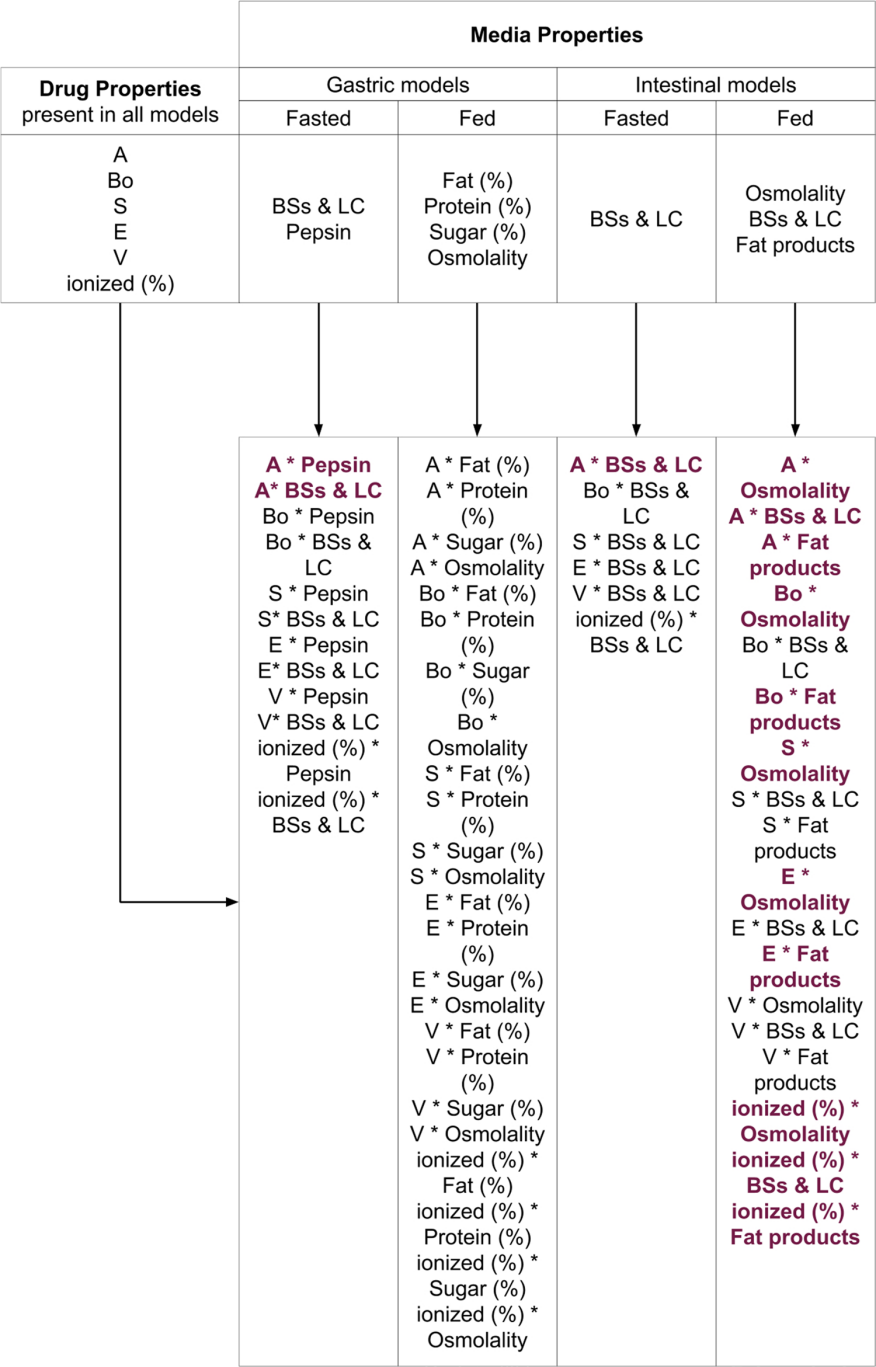
Variables present in each PLS-R model investigated. Drug properties were maintained in every model and media properties were selected according to changes in media composition between pediatric and adult simulated GI fluids. Investigated interactions (between drug properties and media properties) are also described, where interactions coloured in purple represent interactions with VIP values lower than 0.7.

PLS-R analysis was performed with XL-STAT® add-in (Addinsoft, USA) for Microsoft Excel 2016, Office 365® (Microsoft, USA). PLS-R was selected due to its ability to deal with collinearity between independent variables (8). The schematic of independent variables and interactions between variables for each model is shown in Fig. 1. The Sp/Sa (%) was set as the response variable. Each replicate value of drug solubility in pediatric media was used to calculate the ratio of pediatric solubility with reference mean solubility in adult biorelevant media (Sp/Sa (%)). Explanatory variables were set as (i) drug properties, which included the Abraham solvation parameters (Table I); and fraction of ionized molecular species (“ionized (%)”) (obtained from ACD/Labs^©^ 2010–2018), where a positive value was attributed for compounds with a basic pKa, and a negative value for compounds with an acidic pKa; (ii). changes in media when comparing between pediatric and adult biorelevant media (*i.e.* pepsin, bile salts, lecithin, sodium oleate, glyceryl monooleate, fat (%), sugar (%), protein (%), etc.); and (iii). interactions between medium and drug individual variables. Composite variables (combination of two or more individual variables) were used when the ratio of two individual medium components was maintained throughout each pediatric medium. The influence of "Bile salts and Lecithin (BSs & LC)" was evaluated as a composite variable comprised of sodium taurocholate (NaTC) and lecithin concentrations, and the variable "fat products" was set to represent concentrations of sodium oleate and glyceryl monooleate. The influence of interactions between each set of variables (drug and media independent variables) was also investigated.

**Table I:** Drugs Investigated in this Study and Abraham Solvation Parameters (ACD/Absolv. Prediction Module 2016.1)

Compound	logP	pKa (s)	A	Bo	S	E	V
Mesalazine	0.98 (12)	2.3 (acidic) & 5.69 (basic) (12)	0.93	0.74	1.52	1.22	1.09
Montelukast	8.79 (13)	2.7 (basic) & 5.8 (acidic) (13)	0.88	1.68	2.86	3.75	4.49
Nitrofurantoin	-0.47 (14)	7.2 (acidic) (15)	0.24	1.34	2.03	1.65	1.45
Phenytoin	1.92 (16)	8.06 (acidic) (16)	0.44	1.15	2.04	1.94	1.87
Naproxen	3.18 (14)	4.18 (acidic) (17)	0.57	0.76	1.49	1.54	1.78
Indomethacin	4.27 (14)	4.45 (acidic) (18)	0.57	1.26	2.49	2.44	2.53
Dapsone	0.97 (14)	3.2 (basic) (19)	0.45	1.35	1.42	9.71	2.84
Dipyridamole	2.74 (20)	5.7–6.4 (basic) (20)	0.95	3.03	2.95	20.53	2.90
Mebendazole	2.8 (21)	3.5 (basic) (21)	0.71	1.38	1.34	12.82	2.76
Amiodarone	7.57 (22)	8.73 (basic) (22)	0	1.30	1.31	18.09	2.49
Griseofulvin	2.18 (14)	not applicable	0	1.58	1.59	11.78	2.32
Spironolactone	2.26 (14)	not applicable	0	1.63	1.65	16.02	3.81
Carbamazepine	2.45 (14)	not applicable	0.39	0.92	0.94	10.79	2.06
Fenofibrate	5.3 (23)	not applicable	0	1.13	1.14	11.914	2.11

**A** hydrogen-bonding acidity; **B** hydrogen-bonding basicity; **S** dipolarity/polarizability; **E** excess molar refraction; and **V** McGowan characteristic volume

The model quality was evaluated on the square of the coefficient of determination (R^2^) and goodness of prediction (Q^2^). R^2^ and Q^2^ values close to 1 represent a good model fit and a high predictive power, respectively (8). The number of principal components for each model was selected based on the model’s optimum Q^2^ value. A Q^2^ value higher than 0.5 is considered acceptable for good model predictability (8). Independent variables are considered to be significant if Variable Importance in Projection (VIP) values are higher than 1.0, values between 0.7 and 1.0 were considered to have moderate impact on the response variable, whereas VIP < 0.7 are generally regarded as not significant (8). The VIP is a weighted summary of the influence of each individual variable on the PLS model. The VIP is calculated as a weighted sum of the squares of the PLS loadings for each variable, which are representations of the variables impact and its correlations in the model, taking into account the amount of Y-variance explained (9–11)*.*

In the PLS-R analyses, the outliers were identified by comparison of the distance of each observation to the model in the Y-plane (DModY) with their calculated critical value (DCrit(Y)); an outlier is present if the standardized DCritY for an observation is greater than DCritY (9–11). Outliers were only exluded if (i) the PLS model improved significantly after exclusion and (ii) there was a clear scientific rational on why the data should be excluded (*i.e.* why the current models would not be able to explain the results).

## Results

### Fasted gastric simulated fluids

**Fig. 2:**
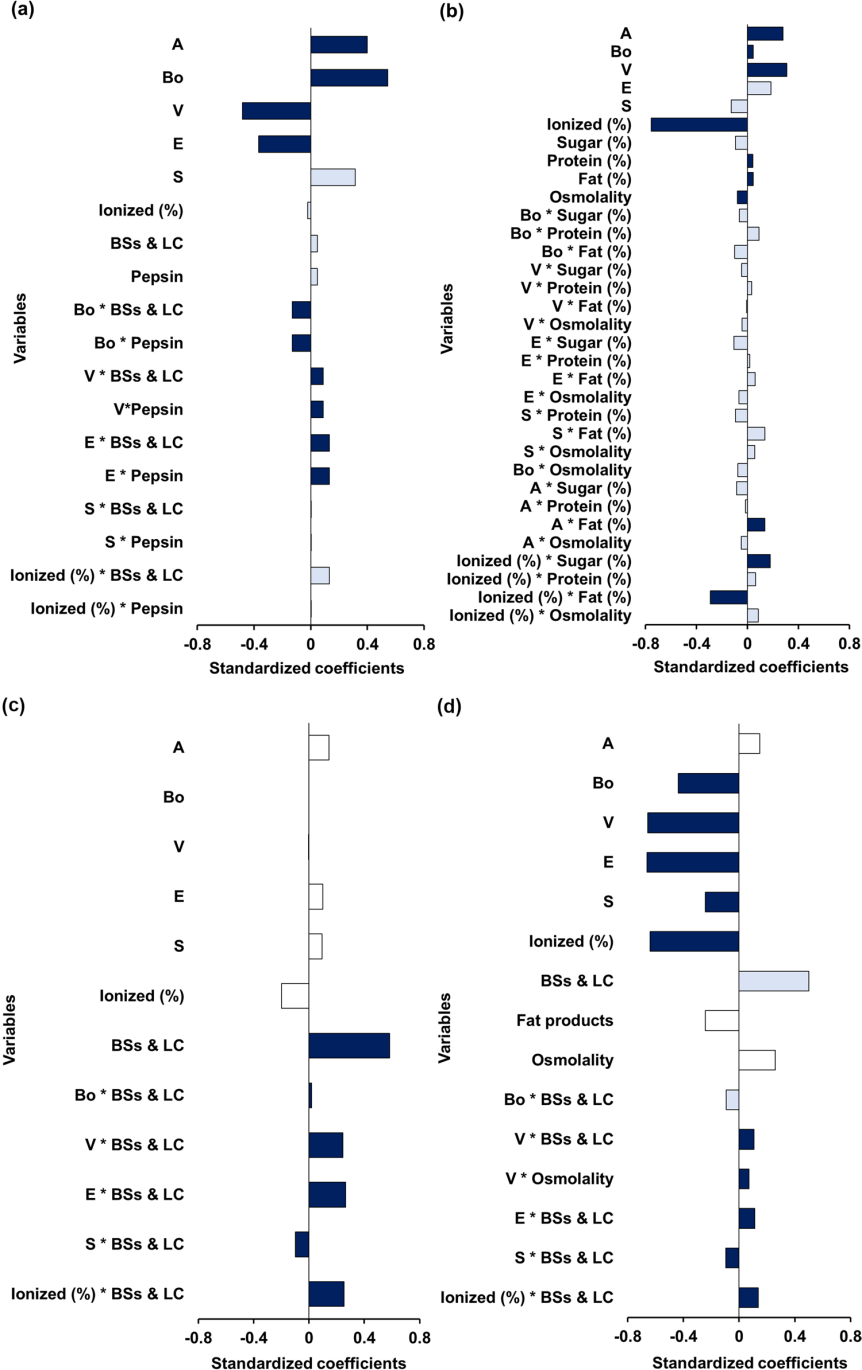
Standardized coefficients in pediatric fasted (a) and fed (b) gastric fluids, and pediatric fasted (c) and fed (d) intestinal fluids corresponding to the variables and interactions investigated in each model. Bars in dark blue denote coefficients of VIP ≥ 1.0 (significant impact), and bars of lighter blue represent variables with 1 ≥ VIP ≥ 0.7 (moderate impact), which are considered to have an effect on the response value Sp/Sa (%).

The PLS-R model for the fasted gastric state is presented in Fig. 2a. The best model had three principal components and presented an R^2^ = 0.560, although it showed limited predictive power (Q^2^ = 0.348). Interactions between media variables (pepsin and BSs & LC) and McGowan volume (V) and molar refraction (E), were significant positive predictors for the Sp/Sa (%), and hydrogen-bonding basicity (B) interactions with BSs & LC and pepsin showed a negative impact on Sp/Sa (%).

### Fed gastric simulated fluids

The model for the Sp/Sa (%) in the fed gastric state was best with four principal components and was able to account for both good Y variability (R^2^ = 0.897) and good predictive power (Q^2^ = 0.768). The model showed that the variables ionized (%) (*i.e.* ionized fraction), hydrogen-bonding acidity, and basicity (A and B respectively) and McGowan volume (V) were the individual factors with the highest effect on Sp/Sa (%) (VIP > 1). Based on the standardized coefficient values, ionized (%) was identified as the most significant factor which showed a negative impact on Sp/Sa (%)(Fig. 2b).

### Fasted intestinal simulated fluids

The PLS-R model for the Sp/Sa (%) observed in fasted intestinal state displayed optimum Q^2^ values with three principal components. The PLS-R model developed for pediatric fasted intestinal state presented an R^2^ = 0.768 and showed a suitable predictive power of Q^2^ = 0.651. Interestingly, the model showed that none of the individual drug properties significantly affected Sp/Sa (%) (Fig. 2c). BSs & LC was the most important variable influencing drug solubility for which a standardised coefficient with a positive value was observed. This explains the increases in Sp/Sa (%) when the concentrations of NaTC and lecithin increase. Interactions between the Abraham solvation parameters and BSs & LC were considered highly significant for identifying solubility changes in pediatric fasted intestinal fluids compared to that of adults.

### Fed intestinal simulated fluids

Montelukast solubility in pediatric fed intestinal simulated fluids was identified by preliminary analysis as outlier (data not shown), and excluded from the PLS-R analysis for the fed intestinal state. This is related to the fact that the Sp/Sa (%) in the fed state intestinal fluids showed values lower than 100% for all drugs, except montelukast; as the pediatric media contain a smaller amount of sodium chloride, the solubility results for montelukast were likely related to a common-ion effect, since the sodium salt of montelukast was used in the experiments. It was observed that the model quality (R^2^ and Q^2^) improved notably after exclusion of montelukast.

The final PLS-R model developed for the Sp/Sa (%) in the fed state intestinal state accounted for an acceptable amount of variability of Y (R^2^ = 0.744) and presented a predictive power of Q^2^ = 0.587. The standardized coefficients showed that all individual drug properties significantly affected Sp/Sa (%), except for the molecular predictor of hydrogen-bond acidity, A (Fig. 2d). Additionally, BSs & LC were important variables affecting Sp/Sa (%). The interactions between the Abraham solvation parameters and BSs & LC were considered to be highly important for identifying changes in drug solubility in pediatric versus adult fed intestinal simulated fluids.

## Discussion

As described previously (Materials and Methods section), the Abraham molecular solvation parameters are commonly used to describe the logarithm of any solvation-related property (SP) that is expressed as a linear free energy relationship of the solute properties (Eq. 1) (3). Previous work used this approach of a linear free energy relationship to predict the ratio of solubility enhancement (log(SE)) in FaSSIF biorelevant medium when compared to solubility in a buffer solution (*i.e.* blank FaSSIF buffer) for a series of apparently neutral drugs (2). Drug solubility enhancement was treated here analogues to a partitioning process; it can be viewed as a drug transfer from a buffer solution to a mixed micellar environment (the FaSSIF medium with bile salts and phospholipids), resulting in increased drug solubilization. The results from the previous study also showed that such solubilization was strongly favoured for compounds with higher values of McGowan volume (V) (2). The influence of molecular size on drug solubility, and its relationship with the surface area of the drug, was shown, as expected, for comparatively lipophilic drugs (2).

In our study, Abraham solvation parameters were used with a broader scope than a single linear free energy relationship for a colloidal medium compared to a buffer. Thus, the Abraham solvation parameters can be used also for other empirical correlations eventually in combination with further molecular predictors, for example, the percentage of ionized drug to go beyond the limitation of apparently neutral compounds. The aim of our analysis was to investigate and understand drug solubilization in pediatric biorelevant simulated fluids. As individual variables, at least one of the Abraham solvation parameters was important for the prediction of differences between drug solubility in adult and pediatric simulated GI fluids, except for the case of the fasted intestinal simulated media. The effect of the basicity parameter was positively correlated to Sp/Sa (%) in the gastric fluids, whereas it had a negative effect in the intestinal media. A negative impact of basicity has previously been described for partitioning into apolar media (3).

Apart from the molecular characteristics, different medium properties were also considered for the PLS-R models of Sp/Sa (%). Since the response in the PLS-R models was the relative solvation between two biorelevant media (*i.e.* Sp/Sa (%)) (and not in comparison to a buffer), one would expect that, theoretically, molecular properties would exhibit only subtle effects when media characteristics, with regards to their molecular environment inside of the mixed micelles, are similar. This expectation was in line with the obtained results in this study, for the fasted gastric state conditions, where adult and pediatric media have the least amount of differences in their composition. The different Abraham solvation parameters demonstrated more pronounced effects on Sp/Sa (%) in the fed state conditions, which was not only due to the higher concentration of the colloidal phase but must have also accounted for changes in the colloidal solvation environment as reflected by compositional differences of the media for the different age groups.

In the fasted gastric state, the Abraham solvation parameters which were the most important factors influencing solubility differences between pediatric and adult simulated GI conditions were excess molar refraction (E), McGowan volume (V), acidity (A), and basicity (Bo).

The fed gastric media is the only set of media where pH is changed between adult medium and pediatric media, therefore, the ionized (%) was observed to be one of the most important variables influencing Sp/Sa (%). Other important variables that influence solubility changes between adult and pediatric media were fraction of ionized molecular species (“ionized” %), McGowan volume (V), acidity (A). The composition of the fed gastric medium was also shown to be an important factor influencing drug solubility in differences in adult and pediatric fed gastric simulated fluids, especially in terms of protein and fat content, and its osmolality. These results suggest that differences in the composition between different types of feeding (*e.g*. formula, cow's milk) can affect the solubility of drugs in GI fluids (7).

In the fasted intestinal state, BSs & LC is the single most important factor influencing changes in drug solubility in pediatric media in comparison to adult medium. Additionally, interactions of this variable with drug properties such as ionized (%), McGowan volume (V) and excess molar refraction (E) were important factors influencing solubility differences between pediatric and adult simulated GI fluids.

In the fed intestinal state, ionized (%), McGowan volume (V), excess molar refraction (E), basicity (Bo) and polarizability (S) were the main individual drug-related contributing factors for changes between drug solubility in pediatric simulated media compared to the adult simulated medium. Moreover, similarly to the fasted intestinal state, interactions of media composition in terms of BSs and LC with drug properties (*i.e.* McGowan volume (V), excess molar refraction (E), polarizability (S) and ionization) contribute to the magnitude of the observed solubility changes between adult pediatric media.

In our previous investigation, a multivariate statistical analysis was also used to better understand drug solubilization as a function of a drug’s physicochemical properties (defined by logP, ionized (%) and molecular weight (MW)) and the composition of gastrointestinal fluids (7). The results revealed the importance of logP and MW, and their interactions with BSs and LC in driving solubility differences between adult and pediatric simulated GI fluids (7). Drug MW is fundamentally related to Abraham solvation parameters such as McGowan volume (V) and excess molar refraction (E). Interestingly, the developed models in this study showed that V and E were found to be influencing variables of Sp/Sa (%), as individual and interacting factors. Therefore, both V and E are considered crucial variables towards the developed PLS-R models with Abraham solvation parameters as drug properties (3).

Niederquell and Kuentz discussed that the approach of using the Abraham solvation parameters for the prediction of solubility enhancement (log (SE)) could be problematic if compounds are not just solubilized either in bulk solution or in the core of colloids (3). Thus, especially ionized drug may also interact with the surface of the mixed micelles in biorelevant media. As the present study did not only consider neutral compounds, fraction of ionized molecular species (%) was taken into account, explicitly. Although surface interactions with the biorelevant media may have occurred in some cases, the present approach proved to be viable. Thus, for modelling and understanding of the factors driving solubility differences between adult and pediatric GI fluids, the selected predictors were shown to perform well in all cases, except for the fasted gastric fluids (3). We hypothesise that this is related to the fact that these pediatric media present the least differences in composition as compared to the adult medium.

## Conclusions

The Abraham solvation parameters were helpful in the discrimination of drug solubilization between adult and pediatric simulated GI fluids. Results of this study showed that the combination of Abraham solvation parameters with ionized (%) was useful as a theory-informed set of molecular predictors. Additionally, our findings suggest that the Abraham molecular parameters were able to identify that the molecular solvation environment in the fasted state was more similar between the pediatric and adult conditions than when compared to differences in the fed state. In the intestinal fasted and fed state, the high relative contribution of the composite variable of BSs & LC to the Sp/Sa (%) suggests that adequate choice of truly age-appropriate medium composition is particularly important for these states. Therefore, further characterization of pediatric GI fluids will support the development of more robust biopharmaceutical *in vitro* tools. Future models may profit from even larger datasets but for the selection of meaningful molecular descriptors, the Abraham solvation parameters hold much promise.
